# Noise Analysis for Correlation-Assisted Direct Time-of-Flight

**DOI:** 10.3390/s25030771

**Published:** 2025-01-27

**Authors:** Ayman Morsy, Jonathan Vrijsen, Jan Coosemans, Tuur Bruneel, Maarten Kuijk

**Affiliations:** Department of Electronics and Informatics, Vrije Universiteit Brussel, Pleinlaan 2, 1050 Brussels, Belgium; jonathan.vrijsen@vub.be (J.V.); jan.matei.coosemans@vub.be (J.C.); tuur.bruneel@vub.be (T.B.); maarten.kuijk@vub.be (M.K.)

**Keywords:** pixel noise analysis, single-photon avalanche diode (SPAD), time-of-flight (ToF), light detection and ranging (LiDAR), depth sensing

## Abstract

The development of a correlation-assisted direct time-of-flight (CA-dToF) pixel provides a novel solution for time-of-flight applications that combines low power consumption, robust ambient shot noise suppression, and a compact design. However, the pixel’s implementation introduces systematic errors, affecting its performance. We investigate the pixel’s robustness against various noise sources, including timing jitter, kTC noise, switching noise, and photon shot noise. Additionally, we address limitations such as the SPAD deadtime, and source follower gain mismatch and offset, identifying their impact on performance. The paper also proposes solutions to enhance the pixel’s overall reliability and to improve the pixel’s implementation.

## 1. Introduction

Time-of-flight (ToF) imaging is an innovative technology that offers promising capabilities for depth mapping. There is increasing interest in developing systems characterized by low power consumption, high image resolution, reliable distance accuracy, and minimal distance precision errors [1]. Such requirements are significant in the automotive industry for machine vision applications and in consumer electronics such as smartphones and laptops [2,3,4]. The most common technologies used for ToF are direct time-of-flight (dToF) and indirect time-of-flight (iToF). A significant amount of research has focused on characterizing each technology’s optimal theoretical and practical performance, while addressing their inherent technological limitations. The sources of error in a ToF camera can generally be classified into two categories: systematic errors and non-systematic errors [5].

Non-systematic errors are associated with stochastic noise and depend on the characteristics of the detected scene. Key contributors to non-systematic errors include illumination and shot noise, material properties such as color and surface finish, and scene properties such as multi-path reflections, scattering, and motion. These errors have been extensively studied, with various mitigation methods proposed [5,6].

On the other hand, systematic errors are relatively stable and typically related to the camera’s internal operation. These errors can arise from factors such as pixel architecture, process variation, readout noise, clock mismatch, switching errors, and unaccounted parasitic elements, which can lead to issues such as distance distortion, fixed pattern noise (FPN), and cyclic distance accuracy errors [5,7]. The nature of systematic errors varies between dToF and iToF technologies.

Direct time-of-flight technology is designed to measure distances by detecting the arrival time of photons, which are recorded to generate a histogram. This histogram may feature one or multiple peaks which are associated with direct or multi-path reflections of the laser source. The main building block of the system is an avalanche detector, with the arrival time converted into digital or analog values [8]. The data points are stored in histogram memory, enabling the accumulation of data from multiple laser cycles to create the histogram. A common dToF architecture employs a single-photon avalanche diode (SPAD) sensor in conjunction with a time-to-digital converter (TDC) [9]. The bit depth and linearity of the TDC directly limit the effective bin width of the histogram, constructed in the digital domain. Besides this, the detection of a photon in a SPAD briefly paralyzes the device, known as SPAD deadtime. This deadtime can lead to distortion of the histogram through the so-called pile-up effect. The relatively large amount of photons associated with a laser peak compared to the background light significantly deteriorates the detection of any incident photons in the deadtime thereafter. Consequently, the mean of the detected peaks may shift toward the beginning of the laser pulse arrival time, degrading the calculated distance accuracy.

A common strategy used to mitigate the impact of deadtime on peak distortion involves combining multiple SPADs with a single TDC per pixel [10,11]. This approach reduces the adverse effects of deadtime, at the expense of increasing the pixel complexity.

Another similar contributor to pile-up is TDC deadtime, which is the duration required for the TDC to convert a detected photon into a signal. Multi-event TDCs have therefore been developed to address TDC pile-up, while simultaneously enhancing the system dynamic range, although these come with increased data rate demands [12,13]. A synchronous summation technique (SST) TDC was proposed to minimize pile-up effects and optimize the dynamic range in [14], in which multiple SPAD signals are combined through logical gates alongside an oversampling TDC. In general, the additional noise sources associated with dToF systems such as SPAD timing jitter and TDC reference clock jitter further compromise the precision of distance measurements.

Indirect time-of-flight technology measures the distance by utilizing a modulated light source to illuminate a scene, in coordination with the shutter of a photonic mixer device as a detector. The time of flight is determined by calculating the phase difference between the modulated shutter and the light reflected from the scene. The light source can be either a pulsed or continuous wave emitter, which can employ sinusoidal or square wave modulation [8]. The photonic mixer is typically a gated CMOS sensor that controls the electric field within the sensor to modulate the detected photo-carriers in relation to the illumination modulation [6].

The performance of the detector is influenced by its ability to modulate charge carriers quickly, its well capacitance, and its dark current. The modulation mismatch between the detector and the illumination leads to a phase error. As a result, calibration is required, to ensure uniform clock arrival time across the entire pixel array [15]. Limitations in well capacitance can cause sensor saturation under high ambient light conditions and reduce sensitivity for long-range detection. Therefore, multiple publications have demonstrated a limited operation range with limited ambient light levels [16,17].

Other iToF noise sources affecting the precision of distance measurements include thermal noise, flicker noise, kTC noise, and ADC non-linearity. These types of noise are typically modeled as Gaussian processes. Various readout schemes have been developed, such as the column-parallel single-slope ADC in [18], and the column delta-sigma ADCs and two-step ADCs in [19,20], respectively.

Recently, a correlation-assisted direct time-of-flight (CA-dToF) pixel architecture has been proposed to mitigate ambient shot noise using an in-pixel exponential moving average circuit [21]. The CA-dToF pixel is designed to address the limitations of current ToF technologies, particularly in terms of reducing power consumption and enhancing image resolution [22]. This design integrates elements of both dToF and iToF pixel operations: it employs a SPAD-based system with a time-to-amplitude converter, similarly to dToF systems, while generating an analog output, as in iToF systems. Despite these innovations, the pixel design faces systematic challenges that affect the overall performance.

The article discusses CA-dToF systematic noise sources and suggests potential solutions. The structure of the paper is as follows: Section 2 introduces the pixel architecture and its operational principles. The SPAD effects on pixel operation are discussed in Section 3. Section 4 examines switching noise sources and their impact on pixel performance. In Section 5, the different analog input signals and their associated noise sources are analyzed. Finally, Section 6 explores potential design optimizations to address these challenges.

## 2. Discussion of the Pixel Architecture

**Figure 1:**
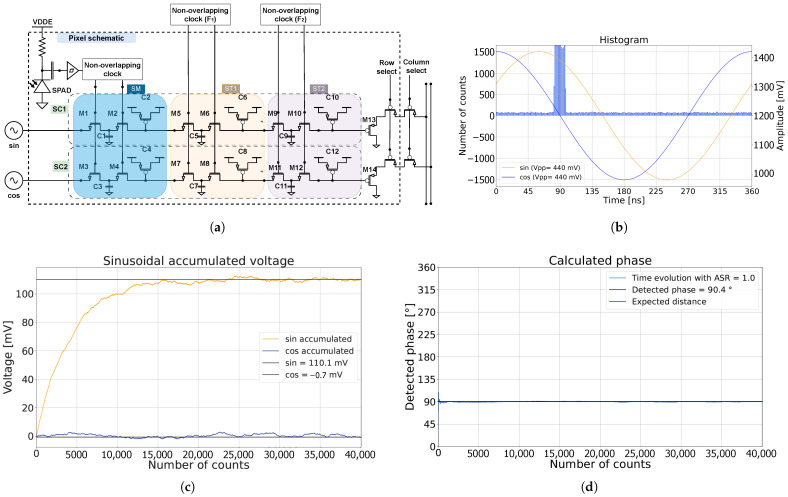
(**a**) The CA-dToF pixel with multiple stages (ST1) and (ST2), externally driven over the full array with frequencies $F_{1}$ and $F_{2}$, respectively [22]; (**b**) histogram of the detected photons without dead-time effect, with the applied sinusoidal signals and ASR = 1; (**c**) the accumulated voltage for the sinusoidal signals; (**d**) the calculated phase as a function of the detected counted triggers. The expected distance overlaps with the detected phase.

As illustrated in Figure 1a, the CA-dToF pixel consists of two channels (SC1 and SC2), each composed of three stages. The sampling stage (SM) samples the sinusoidal inputs (sin: *I* and cos: *Q*) when a photon is detected [22]. Note that different inputs can be used, as demonstrated later in the paper. The SPAD is passively quenched using an electrically isolated high-voltage resistor, and the signal is AC coupled to a low-voltage Schmitt inverter. The hysteresis mitigates afterpulsing, at the cost of sensitivity at high intensity light levels. The output voltage $V_{i}$ is averaged according to an exponential weighted moving average (EWMA), as expressed in Equation (1). The integration length $n_{av}$ depicts the length of the tail of the weighted average. The voltages sampled from the applied input signals are labeled $V_{m,i}$.

(1) $$V_{i}=\lambda \cdot V_{m,i}+\left(1-\lambda \right)V_{i-1}$$

(2) $$\lambda =\frac{1}{n_{av}}=\frac{C_{1}}{C_{1}+C_{2}}=\frac{C_{3}}{C_{3}+C_{4}}$$

Figure 1b demonstrates how sinusoidal inputs *I* and *Q* were sampled by photons in an ideal simulation, under the assumption of zero SPAD deadtime, uniformly distributed ambient light in time, and a laser arrival at $90^{\circ }$ phase shift. The applied sinusoidal signals had an amplitude of 220 mV and an offset of 1.2 V. After cancellation of the offsets, the evolutions of the measured voltages are shown in Figure 1c. The measured voltages $\hat{I}$ and $\hat{Q}$ stabilized and reached equilibrium, aside from random oscillations due to laser and ambient photon shot noise. The random oscillation determined the amplitude precision and decreased as $n_{av}$ increased. In this simulation, $n_{av}$ was 300, with an ambient-to-signal ratio (ASR) of 1. Note that the amplitude of the measured voltages were then attenuated by a factor of 2 due to ambient suppression. The calculated phase of the laser arrival was calculated and is plotted in Figure 1d.

The first and second averaging stages (ST1 and ST2) perform additional sampling and averaging of the voltage, modulated externally via two non-overlapping clocks (F1 and F2), respectively. The parasitic capacitors ($C_{1}$, $C_{3}$, $C_{5}$, $C_{7}$, $C_{9}$, and $C_{11}$) are orders of magnitude smaller than the averaging capacitors ($C_{2}$, $C_{4}$, $C_{6}$, $C_{8}$, $C_{10}$, and $C_{12}$) with an equal ratio of approximately 100. Therefore, stages ST1 and ST2 minimally affect the voltage across the averaging capacitors. It is possible to connect ST1 and ST2 with SM by shorting the switching transistors ($M_{5}$ through $M_{12}$) to form a single switched capacitor stage with an approximate integration length $n_{av}$ of 300. In other words, the pixel can function as a single stage or multiple stages.

The two output voltages are each buffered using a low-threshold PMOS source follower for the readout ($M_{13}$ and $M_{14}$).

The basic pixel operation has already been reported in [22]. However, this previous work did not account for systematic noise sources and their impact on pixel performance. This work identifies key systematic noise contributors and their impacts.

## 3. Consequences of SPAD Non-Idealities on Pixel Operation

In general, quenching of the SPAD can be achieved passively using a resistor, or actively by reducing the voltage across the SPAD to below the breakdown voltage using a transistor.

The passive approach offers a simple, compact, and more energy-efficient circuit, while the active method allows for higher count rates and a reduced afterpulsing probability. As already mentioned, an electrically isolated high-voltage resistor was employed in this work for passive quenching and rearming of the SPAD. Upon triggering, the avalanche current reduces the voltage across the SPAD towards the breakdown voltage. The resistor then recharges the SPAD to $V_{dde}$ within a time frame determined by the RC constant of the quenching resistor and the capacitance at the cathode node. Due to technology constraints, a high-voltage AC coupling capacitor was required between the high-voltage cathode and the low-voltage sensing circuit. In this section, the noise associated with the SPAD circuit, illustrated in Figure 1a, is discussed.

### 3.1. The Effect of Internal Timing Jitter

When a photon is incident on the SPAD it may generate an electron–hole pair within the diode’s junction. If this occurs in the multiplication region, the resulting carriers are rapidly accelerated by the strong electric field, triggering an avalanche. However, carriers generated outside this region must diffuse into it. Diffusion is a slow and stochastic process compared to the drift within the field, causing a delayed response. The location of carrier generation thus contributes to timing jitter. Since photon absorption is probabilistic, penetration depths vary following the Beer–Lambert law. Therefore, jitter arises not only across wavelengths but also within a single wavelength.

In the circuit illustrated in Figure 1a, the SPAD jitter propagates through the sensing Schmitt inverter and the non-overlapping clock generation. The timing jitter $\sigma_{jitter}$ results in a variation in the measured voltage after the SM stage. The relation is proportional to Equations (3) and (4), in which $\hat{I}$ and $\hat{Q}$ are the measured voltage at equilibrium for the sine and cosine signals, respectively. The effect of timing jitter on the variation in the measured voltage at equilibrium is significantly reduced due to the integration length $n_{av}$ and is negligible compared to the variation resulting from photon shot noise and laser jitter.

(3) $$\sigma_{\hat{I}}∼\frac{\sigma_{jitter}}{\sqrt{2n_{av}-1}}$$

(4) $$\sigma_{\hat{Q}}∼\frac{\sigma_{jitter}}{\sqrt{2n_{av}-1}}$$

### 3.2. The Effect of the Bias Voltage

The excess bias voltage of the SPAD is $V_{ex}=V_{dde}-V_{bd}$ when the voltage across the SPAD is $V_{dde}$, where $V_{bd}$ is its breakdown voltage. The likelihood of triggering due to an incident photon is defined as the photon detection probability (PDP). Note that a higher $V_{ex}$ increases the PDP but also raises the dark count rate (DCR). In the proposed system, dark counts are effectively rejected as if they were ambient photon counts, resulting in an additional shot noise component in the measured voltage. To achieve the best signal-to-noise ratio (SNR), the bias voltage $V_{dde}$ should be carefully selected.

**Figure 2:**
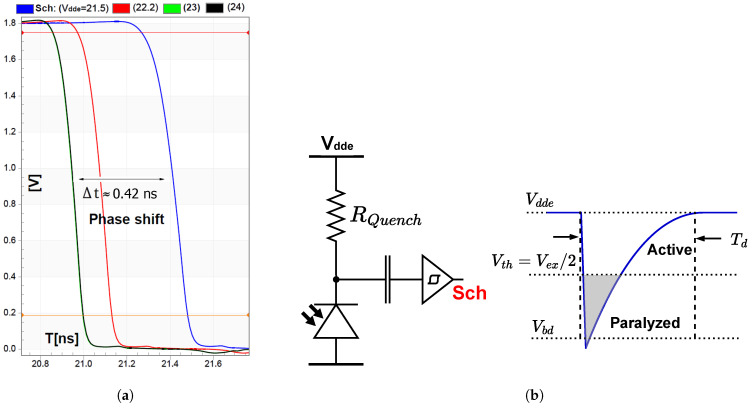
(**a**) The response of the inverter with different $V_{dde}$ in a simulation when $R_{Quench}$ = 500 kΩ and with an overall SPAD and AC coupling capacitance of around 14 fF, showing a propagation delay up to 0.42 ns and varying fall-times; (**b**) the paralyzing effect of the inverter after a SPAD trigger. In our implementation, $V_{bd}$ = 19.6 V.

During normal operation of the pixel, the voltage across the SPAD may be $V_{dde}$ when it is fully armed, or $V_{bd}$ when is fully quenched, or anywhere in between when it is rearming. During a trigger event, the voltage across the SPAD rapidly drops by the instantaneous $V_{ex}$. This drop is coupled towards the sensing node via the coupling capacitor. If the SPAD was fully armed, the drop is guaranteed to surpass the threshold $V_{th}$ of the inverter, resulting in a low propagation delay. However, a smaller drop may barely exceed the threshold, resulting in a significant propagation delay and introducing delays in the non-overlapping clock generation, and thus in the sampling stage. These delays cause consistent phase shifts in the detected arrival time. Figure 2a shows the delay as $V_{ex}$ varies. The maximum phase delay was 0.42 ns in the simulations.

The usage of an inverter inherently limits the detection of photons with $V_{ex}$ lower than $V_{th}$, as presented in Figure 2b. The sensing circuit is then paralyzed, although the SPAD can still detect photons at a reduced PDP in this region. Essentially, the inverter effectively suppresses afterpulsing, albeit at the cost of limiting the sensitivity.

Increasing $V_{dde}$ somewhat mitigates this effect by reducing the overall recharging time towards a sufficient excess bias, ensuring that most trigger events surpass the threshold. As shown in Figure 2a, no significant delay was observed when $V_{dde}$ was set to 23 V or 24 V. Therefore, the $V_{dde}$ of 23 V was chosen throughout the pixel characterization.

### 3.3. Deadtime Shadowing Effect

As discussed in the previous section, a consecutive trigger can only be detected by the Schmitt inverter once $V_{ex}$ reaches beyond the paralyzed zone. Hence, the deadtime of the SPAD combined with the sensing circuitry is defined by the quenching time and the time it takes to recharge the SPAD beyond the paralyzed zone of the Schmitt inverter. To illustrate the effect of deadtime on the system confidence, accuracy, and precision, simulations were executed at various deadtimes. The confidence is defined as in Equation (5), where C is the sinusoidal signal confidence without ambient light [22]. This indicates the applied ASR and can be used in further image processing to improve the system’s performance.

(5) $${\hat{C}}_{simulation}=\sqrt{{\hat{I}}^{2}+{\hat{Q}}^{2}}$$

**Figure 3:**
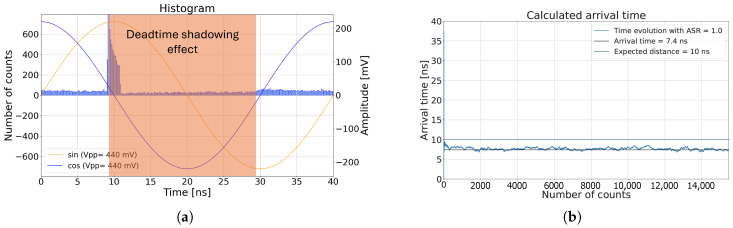
Simulation results demonstrating the shadowing effect with a deadtime of 20 ns. Panel (**a**) shows the histogram of the detected laser pulse with a round-trip time of 10 ns and a pulse width of 1.7 ns. The simulation was conducted over 8,000 cycles and a full detection range of 40 ns. Panel (**b**) shows the detected phase. Due to the deadtime, the detected ambient triggers were no longer uniformly distributed over time, leading to errors at certain ASRs [22].

As noted in [22], a significant shadowing effect may occur following the arrival of the laser pulse, causing the SPAD to predominantly remain in deadtime, due to the high photon levels after the pulse’s arrival. This shadowing effect is further illustrated in the histogram in Figure 3a and the detected phase in Figure 3b. The asymmetric effect distorts the uniform distribution of ambient triggers, adversely impacting both the measured phase and the confidence.

**Figure 4:**
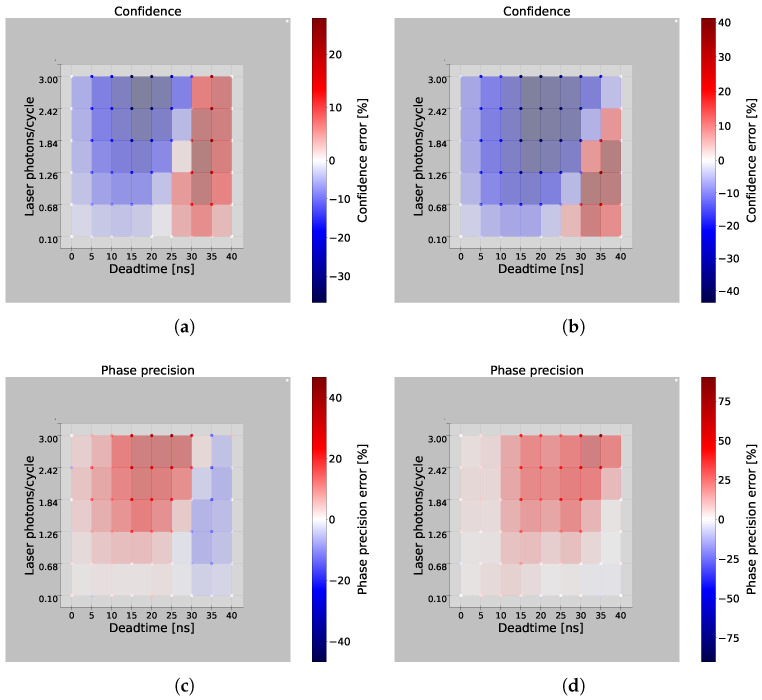

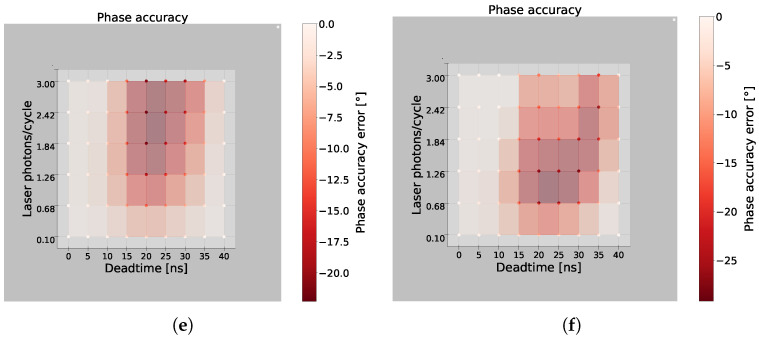
Color maps illustrating the confidence (**a**,**b**), phase precision (**c**,**d**) and phase accuracy (**e**,**f**) at ASR ratios of 1 (left) and 3 (right), respectively. The simulation was performed for deadtimes varying from 0 ns to 40 ns and different laser photons per cycle, varying from 0.1 to 3 laser photons/cycle. The deadtime clearly had an effect on the system’s performance.

In Figure 4, color maps depict the simulated confidence and phase error for an ASR of 1 (left) and 3 (right), for a varying deadtime and a varying number of laser photons per cycle. The non-varying parameters used in the simulation were the same as in Figure 3. The analytical model of the system developed in [22] was used to comprehend the deadtime shadowing effect, as mentioned in Appendix B. The ${\hat{C}}_{analytical}$ follows Equation (A8), where C = 275 mV.

The analytical phase accuracy $E{\left[\theta \right]}_{analytical}$ and precision $\sigma_{\theta \_analytical}$ followed Equations (A11) and (A12), respectively. Each data point in the simulation was averaged over 300 simulation runs, calculating the confidence mean ${\hat{C}}_{simulation}$, the phase mean $\theta_{simulation}$, and the precision $\sigma_{\theta \_simulation}$. The confidence error is defined as the relative deviation of the simulated confidence compared to the analytical confidence, formalized in Equation (6). Similarly, the phase precision error is formalized in Equation (7).

(6) $$\delta_{c}\left[\%\right]=\frac{{\hat{C}}_{analytical}-{\hat{C}}_{simulation}}{{\hat{C}}_{analytical}}\cdot 100\%$$

(7) $$\delta_{p}\left[\%\right]=\frac{\sigma_{\theta \_analytical}-\sigma_{\theta \_simulation}}{\sigma_{\theta \_analytical}}\cdot 100\%,$$

The phase accuracy error is defined in Equation (8), in which $E{\left[\theta \right]}_{analytical}$ and $\theta_{simulation}$ denote the corresponding expected values of the phase and the simulation result, respectively.

(8) $$\Delta \theta =E{\left[\theta \right]}_{analytical}-\theta_{simulation}$$

In an ideal scenario, with a deadtime of 0 ns, the shadowing effect would not impact the confidence, phase precision, or phase accuracy. Increasing the deadtime while maintaining a low number of laser photons per cycle, e.g., at 0.10, significantly reduced the errors in both phase and confidence, due to a diminished shadowing effect. However, increasing the incident laser photons per cycle amplified the shadowing effect, affecting all three error metrics. An oscillation is visible in all color maps, resulting from the deadtime relative to the total cycle duration. In Figure 4a, the confidence error initially became negative with increasing deadtime. For instance, at a deadtime of 10 ns, as shown in Figure 2a, there were fewer triggers between 10 ns and 20 ns. After 20 ns, ambient photons were more likely to trigger events, since the SPAD was outside of deadtime, leading to more ambient triggers when both sinusoidal waves were negative, resulting in a negative deviation from the analytical confidence. With further increases in deadtime, e.g., at 35 ns, this asymmetric effect resulted in more ambient triggers when the sinusoidal waves were positive, thus reversing the sign of the confidence error. As the deadtime approached the cycle width of 40 ns, the shadowing effect diminished again, because the extended deadtime limited the detected photons to approximately one photon per cycle. Therefore, the SPAD was generally ready to trigger before the next laser pulse arrived, mitigating the deadtime shadowing effect.

Increasing the ASR from 1 to 3 caused a general downward shift in the confidence error, due to the higher prevalence of ambient photons. Now, these ambient photons significantly shadowed the laser photons, resulting in fewer triggers in the positive regions of the sinusoidal waves, leading to a more negative deviation from the analytical model.

In Figure 4c,d, $\delta_{p}$ increased with the deadtime, peaked at 15 ns, and then decreased. A positive $\delta_{p}$ indicates that the simulated phase variation was less than the analytical phase. The standard deviation of the phase, $\sigma_{\theta }$, can be approximated as follows [22]:

(9) $$\sigma_{\theta }\approx \sqrt{\left(\frac{E{\left[\hat{Q}\right]}^{2}}{{\left(E{\left[\hat{I}\right]}^{2}+E{\left[\hat{Q}\right]}^{2}\right)}^{2}}\sigma_{\hat{I}}^{2}+\frac{E{\left[\hat{I}\right]}^{2}}{{\left(E{\left[\hat{I}\right]}^{2}+E{\left[\hat{Q}\right]}^{2}\right)}^{2}}\sigma_{\hat{Q}}^{2}\right)}$$

Here, $E[\hat{I}]$ and $E[\hat{Q}]$ are the expected values of the voltages $\hat{I}$ and $\hat{Q}$, respectively, and $\sigma_{\hat{I}}$ and $\sigma_{\hat{Q}}$ are the correspondent standard deviations. According to Equation (9), $\sigma_{\theta }$ is inversely proportional to the square of the confidence defined in Equation (5). Therefore, as confidence increases, phase variation decreases, and vice versa.

Regarding the phase accuracy, Figure 4e shows a significant deterioration around a deadtime of 20 ns. Since the phase is derived from sinusoidal wave measurements, any error in these measurements affects the phase accuracy. At a 20 ns deadtime, the shadowing effect reduced the triggers within the first 20 ns after the laser pulse arrival, causing the negative part of the cosine signal to be shadowed. This resulted in an imbalanced output voltage and thus an accuracy error in the phase, as illustrated in Figure 3.

**Figure 5:**
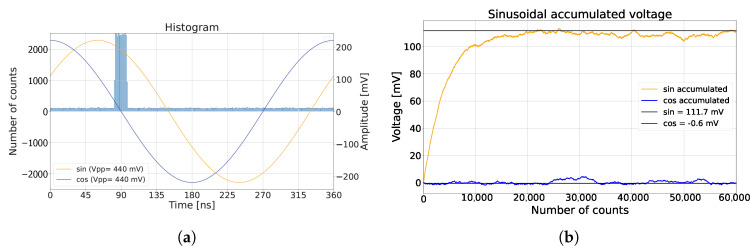

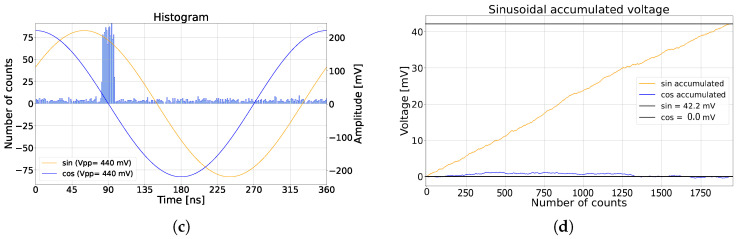
(**a**) The ideal pixel operation with a detection probability of 3 signal photons per cycle in the absence of deadtime with the voltage reaching equilibrium presented in (**b**) for an ASR of 1. (**c**) when the detected photon probability was 0.1 signal photons per cycle, corresponding to the voltage being unable to reach equilibrium, as presented in (**d**).

The phase precision was reduced with a greater integration length $n_{av}$. To achieve more robust averaging, one could simply increase $n_{av}$ at the cost of an increased settling time before reaching equilibrium. This trade-off is demonstrated in Figure 5, where $n_{av}$ = 4000. In Figure 5a,b, there is an abundance of detected photons and the measured voltage reached an equilibrium in a timely manner. In Figure 5c,d, the so called inertia effect was too high for the low number of detected photons, which together with the ASR determined the average time before settling. There was a trade-off between the system’s ability to reject ambient triggers and the number of triggers needed to achieve equilibrium in the output.

Although it is difficult to quantify, the inertia effect significantly influences the system confidence and phase precision. However, it does not always significantly affect the detected phase. The reason is that the phase information is coded in the ratio between the two voltages, not the absolute value of the detected voltages.

The deadtime shadowing effect and inertia effect could be eliminated by implementing a macro pixel of multiple SPADs integrating into one sampling stage SM.

## 4. Temporal and Spatial Noise Contributions of Electronic Circuitry

This section discusses the temporal and spatial noise present due to the in-pixel electronic circuitry. The noise analysis is limited to one sampling stage (SM) only, without ST1 and ST2 being active.

### 4.1. kTC Noise

Every time a SPAD triggers, the reference signal is sampled through $M_{1}$. Due to the finite channel conductance of this sampling switch, the sampled voltage is subject to kTC noise. The noise power in the voltage domain is given by Equation (10), in which $k_{B}$ is the Boltzmann constant and *T* the temperature.

(10) $$\sigma_{{\mathrm{kTC}}_{1}}^{2}=\frac{k_{B}\cdot T}{C_{1}}$$

One can thus compute that the standard deviation $\sigma_{{\mathrm{kTC}}_{1}}$ due to kTC noise between each sample on the sampling capacitor $C_{1}=0.4\;\mathrm{fF}$ is 3.2mV at T=300K. To understand the kTC noise influence over the sampled voltage, the accumulated voltage $V_{i}$ is derived. After rewriting Equation (1) in its closed form, Equation (11) is obtained.

(11) $$V_{i}=\sum_{j=0}^{i}\lambda {\left(1-\lambda \right)}^{i-j}V_{m,j}$$

From Equation (11), the stochastic temporal noise source on the switched capacitor is substantially suppressed by using the averaging capacitor $C_{2}$. One can find the kTC noise on $C_{2}$ in the voltage domain due to $M_{1}$, assuming complete charge transfer through $M_{2}$, equal to Equation (12) converging to Equation (13) in equilibrium [23].

(12) $$\sigma_{M_{1},C_{2},i}^{2}=\sum_{j=0}^{i}\lambda^{2}{\left(1-\lambda \right)}^{2(i-j)}\sigma_{\mathrm{kTC},\;C_{1}}^{2}$$

(13) $$\sigma_{M_{1},C_{2}}^{2}=\frac{\lambda^{2}}{2\lambda -\lambda^{2}}\sigma_{\mathrm{kTC},\;C_{1}}^{2}\approx \frac{\lambda }{2}\sigma_{\mathrm{kTC},\;C_{1}}^{2}=\frac{k_{B}\cdot T}{2(C_{1}+C_{2})}$$

Hence, even though a small sampling capacitor $C_{1}$ is subject to a large kTC noise contribution in the sampled voltage, its approximate power on the accumulated voltage over $C_{2}$ is attenuated by λ/2. For $C_{2}=200\;\mathrm{fF}$, one finds that the standard deviation $\sigma_{M1,\;C_{2}}$ due to kTC noise is 0.10 mV at T=300K.

It is noted that the foregoing analysis can be extended to any white noise source on the sampled voltage, including the thermal noise to which reference signals are subject. The EWMA decreases its power by a factor λ/2.

Furthermore, if the switch $M_{2}$ is conductive during readout, one can show that the noise power spectrum due to its non-zero on-resistance $R_{on}$ is given by Equation (14). In this derivation, it is assumed that the $R_{on}$ contributes a noise power spectrum of $4kTR_{on}$ in series, as analyzed in [23]. By calculating the current from $C_{1}$ to $C_{2}$ in the Laplace domain, Equation (14) is achieved.

(14) $${\bar{v}}_{M2,\;C_{2}}^{2}\left(s\right)\approx {\left|\frac{\lambda }{1+\lambda R_{on}C_{2}s}\right|}^{2}4kTR_{on}$$

Evidently, this is negligible in practice due to the low value of $R_{on}$. However, the switch’s state is not fixed during readout, when only a single sampling stage is utilized. High switching noise would likely be observed in the measurement. This concern is resolved in Section 4.3.

### 4.2. Switch Parasitics

Each voltage sample is subject to charge injection and clock feedthrough caused by $M_{1}$ and $M_{2}$. The effect of these switching parasitics on the measurement is of interest. This discussion limits itself to charge injection—the analysis and conclusions are straightforwardly extended to clock feedthrough.

The residual charge present in the measurement added per sampling iteration is first studied analytically. The channel charge of $M_{2}$ in the ON-state before the detection of the next photon, neglecting the body effect, is given by Equation (15).

(15) $$Q_{ch,2}=WLC_{ox}\left(V_{DD}-V_{i-1}-V_{th}\right)$$

Assuming that the channel charge is divided equally between the source and drain when a switch is turned off, the resulting charge moved to $C_{2}$ is given by $\Delta Q_{2,i-1}=Q_{ch,2}/2$. Analogously, the sampled voltage $V_{m,i}$ is distorted according to Equation (16).

(16) $$\Delta Q_{1,i}=\frac{Q_{ch,1}}{2}=\frac{WLC_{ox}\left(V_{DD}-V_{m,i}-V_{th}\right)}{2}$$

Finally, turning $M_{2}$ back on requires a charge $Q_{ch2}=WLC_{ox}\left(V_{DD}-V_{i}-V_{th}\right)$. Hence, the residual charge left to distort the measurement due to the charge injection per sample is given by Equation (17).

(17) $$\Delta Q_{i}=\Delta Q_{2,i-1}+\Delta Q_{1,i}-Q_{ch2}=\frac{WLC_{ox}}{2}\left(-V_{i-1}-V_{m,i}+2V_{i}\right)$$

The total distortion after *n* samples is given as $\Delta Q_{tot,n}=\sum_{i=0}^{n}\Delta Q_{i}$, for which the expected value in equilibrium is computed in Equation (18).

(18) $$\begin{array}{cc} E[\Delta Q] & =\sum_{i=0}^{\infty }\frac{WLC_{ox}}{2}\left(-\sum_{j=0}^{i-1}\lambda {\left(1-\lambda \right)}^{i-j}E\left[V_{m}\right]-E\left[V_{m}\right]+2\sum_{j=0}^{i}\lambda {\left(1-\lambda \right)}^{i-j}E\left[V_{m}\right]\right)\\ \; & =\frac{WLC_{ox}}{2}E\left[V_{m}\right]\sum_{i=0}^{\infty }\left(-1+\sum_{j=0}^{i}\lambda {\left(1-\lambda \right)}^{i-j}\right)=0 \end{array}$$

This implies that the injection of the charges into the switches’ channels does not cause a distortion in the measured output voltage. Including the dependency of $V_{in}$ on $V_{th}$ in Equation (17) yields the same conclusion. A similar analysis can be performed for the capacitive clock feedthrough.

**Figure 6:**
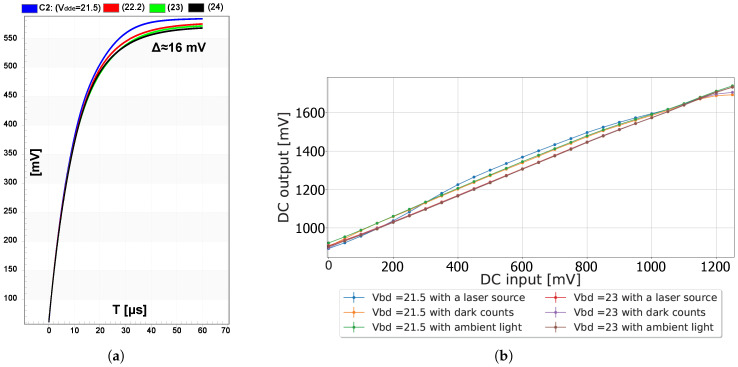
(**a**) The simulated output transients of the sampling stage for different excess bias voltages, when sampling a DC voltage of 500 mV instead of sampling a sinusoidal signal; (**b**) the experimental measurements when sweeping this DC voltage for two different excess biased voltages and for different illumination conditions. The simulation and experimental results show the non-linear repose of the switched capacitor for different SPAD $V_{dde}$, suggesting that switch parasitic noise influenced the switched capacitor performance. However, by applying a $V_{dde}$ higher than 23 V, the switched capacitor response was linear, with minimized switch parasitic noise.

However, the assumption that switching charges are injected equally among the source and drain of $M_{2}$ and $M_{3}$ often does not hold in practice [24]. Hence, it is anticipated that some nonlinear distortion will be present in practice, for example, as the result of the varying fall times, as shown previously in Figure 2. The effects of charge injection and clock feedthrough mismatch are presented in Figure 6a. Notice that when $V_{dde}$ is larger than 23 V, no variation is observed. This was confirmed experimentally when characterizing the DC response of the switched capacitor, as presented in Figure 6b. It was observed that lowering $V_{dde}$ led to different voltage outputs, dependent on the illumination conditions. For a higher $V_{dde}$, a linear response was observed, independently of the illumination condition.

### 4.3. Multi-Stage Sampler

It was shown in [22] that the precision of CA-dToF decreases with increasing ASR. Initially, it was thought that this could be dealt with through increasing $n_{av}$. However, this requires large in-pixel capacitors, limiting both the fill factor (FF) and scalability. In this section, an alternative approach is introduced in the form of multi-stage sampling.

Instead of the single-stage pixel illustrated in the previous work, a three-stage pixel is illustrated in Figure 1a. The addition of devices $M_{5}$, $M_{6}$, and $C_{5}$ gives rise to another EWMA stage ST1.

**Figure 7:**
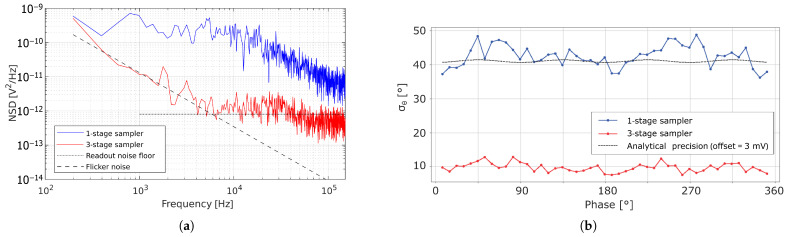
(**a**) The output voltage NSD for a single-stage and three-stage sampler; (**b**) the phase precision when ASR = 16.

This first stage ST1 is driven by an externally applied global sampling pulse F1. This allows increasing the system’s averaging factor in a multiplicative manner. By operating the two additional stages ST1 and ST2 with modulation frequencies of F1 = 1 MHz and F2 = 10 kHz, the switching noise is significantly reduced. The noise spectral density (NSD) for a single-stage and three-stage sampler is illustrated in Figure 7a. Whereas the single-stage sampler suffers from a high broadband noise contribution due to the switches’ distortion during readout, the three-stage sampler exhibits a lower spectrum for voltage-domain readout of pixels, predominantly caused by the source followers $M_{13}$ and $M_{14}$ in Figure 1a.

The use of multiple stages further suppresses the detected photon shot noise, enhancing the phase detection precision, without affecting the phase accuracy or significantly increasing the pixel array power consumption. This improvement is experimentally demonstrated in Figure 7b, where an ASR = 16 was applied.

### 4.4. Fully-Differential Readout

**Figure 8:**
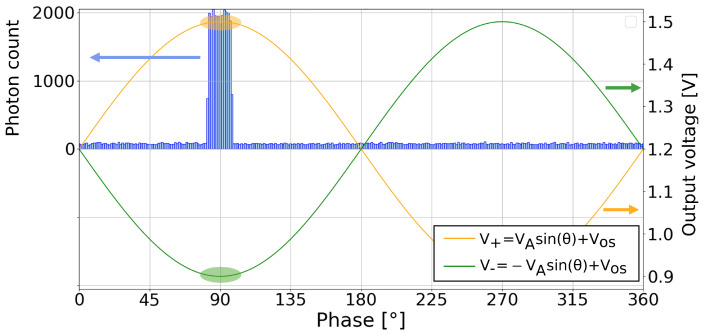
Differential measurement for CA-dToF: measured voltage in non-inverting and inverting channels.

The measured voltages are read out in the voltage domain. This introduces an offset, which varies between pixels due to source follower mismatch, as well as other offsets introduced in the readout pipeline. A classical CMOS image sensor (CIS) accommodates for these offsets through techniques involving delta double sampling (DDS). However, the two channels in this pixel allow for an alternative offset cancellation using differential measurements. For each measurement, two channels referred to as the non-inverting $V_{+}$ and inverting channel $V_{-}$ were devised, receiving the original signal and a signal in anti-phase, respectively. This approach is illustrated in Figure 8. If both channels have a common readout path, a sinusoidal signal free from offset is computed by simple averaging.

(19) $$V_{OS}=\frac{V_{+}+V_{-}}{2}$$

It is shown in Figure 7a that low-frequency noise, which could correspond to time-varying threshold variations in the readout path, is the cause of a significant portion of the total measured noise. Similarly to CISs, which employ correlated double sampling (CDS) [25], the differential readout approach presented here also allows eliminating most low-frequency noise contributions if both samples are sufficiently correlated in time, omitting the need to fully reset the pixel before readout.

## 5. Analysis of the Pixel Performance

In this section, the error associated with the pixel input signal is investigated, and the usage of triangular signals instead of sinusoidal signals as the correlated signal is proposed.

### 5.1. Amplitude Mismatch and Offset Error Effects

**Figure 9:**
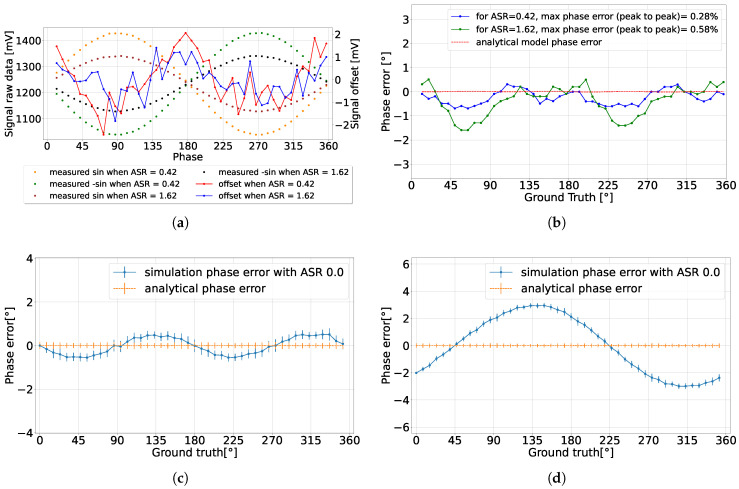
(**a**) The raw measured sinusoidal voltage and the calculated offset, which oscillates with an amplitude of 2 mV, for two different ASRs; (**b**) the phase error associated with the measured offset oscillation [22]; (**c**) the resulting phase error due to a sinusoidal amplitude mismatch of 5 mV when the measured sinusoidal amplitude was 275 mV, or a 2% amplitude mismatch; (**d**) the resulting phase error due to a sinusoidal offset mismatch of 10 mV.

The measured voltages $\hat{I}$ and $\hat{Q}$ are influenced by the system ASR, as well as the gain and offset of the source followers for each channel. The phase information is encoded in the ratio of these two measured voltages. However, source follower gain mismatch causes a periodic oscillation in the calculated phase error, as demonstrated in Figure 9c. The detected confidence $\hat{C}$ and the degree of gain mismatch determine the magnitude of the phase error oscillations. For a fixed amplitude mismatch, a lower detected confidence results in more pronounced phase error oscillations.

Another error associated with the source follower is the offset mismatch. If the offset is not canceled from the detected voltages, this leads to accuracy degradation, as illustrated in Figure 9d. Offset error influences the phase error more than the amplitude mismatch, particularly for low detected confidences. Differential detection, introduced in Section 4.4, is applied to measure and compensate for the source follower offset, reducing the offset-related errors. However, as illustrated in Figure 9a, the signal offset is still not constant across the full detection range, leading to oscillation in the detected phase. This offset oscillation could be attributed to the simplified implementation using only NMOS transistors as switches instead of transmission gates. It is possible that the switches are operating at lower conductance due to insufficient overdrive, depending on the voltages at their drain and source terminals, and thus depending on the input and output voltages of the switched capacitors. In particular, when the sinusoidal signals are sampled at their peaks, this could result in incomplete charge transfer. Variations in switching performance over the full detection range could cause oscillations in the offset.

The offset error is also dependent on the detected amplitude, due to ASR, as shown in Figure 9a. Lower detected amplitudes are associated with reduced offset errors, which in turn minimize the overall offset error. Calibration techniques can be employed to partially address these issues by analyzing the DC response of each source follower and matching the detected amplitude to the gain across the channels in each pixel. The experimental results indicated that this calibration method improved the phase accuracy under low ASR conditions, though it had minimal impact under high ASR conditions. The combined effects of amplitude mismatch and offset error are evident in the experimental data shown in Figure 9b, where phase error oscillations are observed as a result of both phenomena [22].

### 5.2. Sinusoidal and Triangular Input Signals

Up till now, the sinusoidal signal has been employed as a correlation signal to extract distance information. However, the pixel is not limited to sinusoidal signals: it can function using any signal with the same properties as the sinusoids with regards to periodicity, symmetry, and piecewise monotonicity. For instance, triangular signals or square signals can be utilized for signal correlation and ambient light suppression. Among these, a triangular signal offers a compelling alternative due to its simplicity of on-chip implementation, linear performance, and relatively low distortion compared to sinusoidal signals. This study evaluated the performance of triangular signals and compared it to that of sinusoidal signals.

#### 5.2.1. Triangular Amplitude

Building on the mathematical framework established for sinusoidal signals in [22], an analytical model was derived for triangular signals. General formulas for the applied triangular signals can be written as Equations (20) and (21) with period (*T*) and amplitude (*C*).

(20) $$I_{tri}=C\cdot \arcsin\left(\sin\left(\frac{2\pi }{T}t\right)\right)$$

(21) $$Q_{tri}=C\cdot \arccos\left(\cos\left(\frac{2\pi }{T}t\right)\right)$$

Constructing a unified formula for the mean and variance of the detected voltage of the triangular signal proved complex. To address this, an approximate model was developed by segmenting the triangular signal into four distinct regions based on the laser arrival time (*l*), the full-width at half-maximum (*a*) (FWHM), a uniform uncorrelated ambient photon rate (*A*), and a correlated laser photon rate (*S*).

The normalized photon probability distributions for the ambient-only and the ambient-and-laser regions are given by Equations (22) and (23). By integrating the segmented triangular signals over the full range of detection, the expected value of the measured signals $E\left[{\hat{I}}_{tri}\right]\left(l\right)$ and $E\left[{\hat{Q}}_{tri}\right]\left(l\right)$ are presented in Equations (A1) and (A2) in Appendix A.

(22) $$f\left(t\right)dt=\frac{A}{S\cdot a+A\cdot T}dt$$

(23) $$f\left(t\right)dt=\frac{A+S}{S\cdot a+A\cdot T}dt$$

The system’s confidence is once again defined as a measure of the detected amplitude of the triangular signals, which is approximated in Equation (24) for a short laser pulse width (*a*). The definition of the ASR remains unchanged, and can be approximated from the measured confidence according to Equation (25). As for a sinusoidal signal, triangular signal confidence can also be used to detect ASR when the system reaches equilibrium [22].

(24) $$\begin{array}{c} {\hat{C}}_{tri}=E\left[\hat{I_{tri}}\right]\left(l\right)+E\left[\hat{Q_{tri}}\right]\left(l\right)\approx \frac{S\cdot a}{S\cdot a+A\cdot T}\cdot C \end{array}$$

(25) $$\begin{array}{c} \mathrm{ASR}=\frac{A\cdot T}{S\cdot a}\approx \frac{C-\hat{C}}{\hat{C}} \end{array}$$

**Figure 10:**
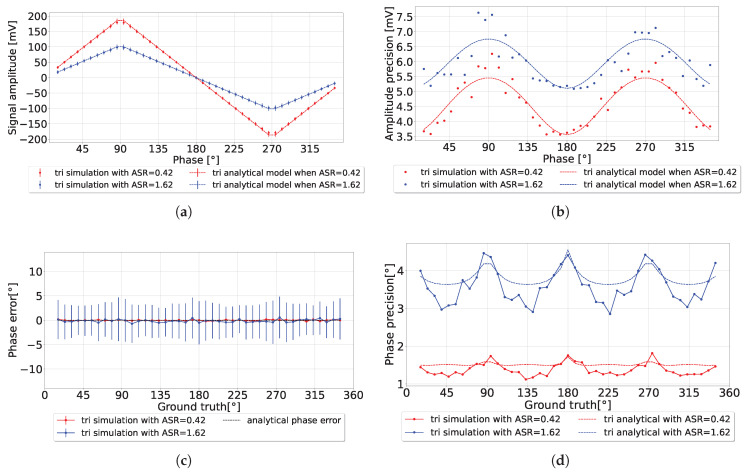
(**a**) Comparison between the simulation results of triangular amplitude for different ASRs; (**b**) the amplitude precision; (**c**) the phase error; (**d**) the phase precision.

**Figure 11:**
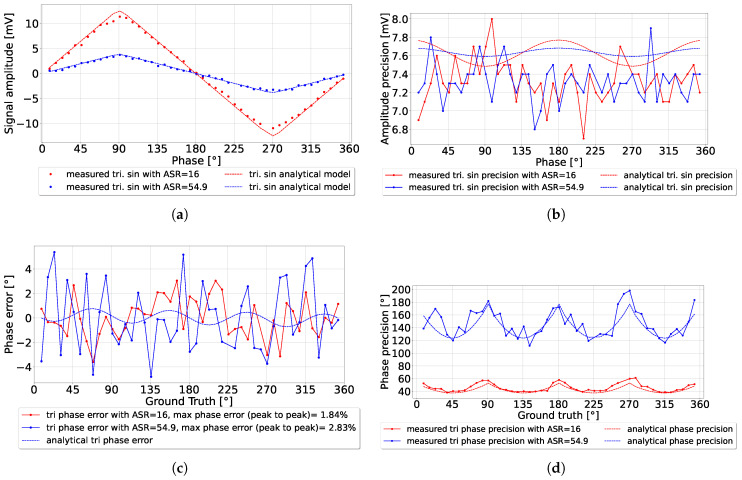
(**a**) Comparison between experimental measurements of triangular amplitude and the analytical model for different ASRs; (**b**) the amplitude precision; (**c**) the phase error; (**d**) the phase precision. The phase error result when ASR = 54.9 showed the robustness of CA-dToF against ambient light.

The system was simulated in Python to test the system’s operation over the full range of detection for a fixed ASR. The simulation parameters were chosen to match the experimental parameters. Both utilized a laser pulse *a* = 1.7 ns with a detection period *T* = 40 ns. Every data point was simulated 200 times to measure the average amplitude and precision. The integration length $n_{av}$ = 300 as a single stage, with applied amplitude *C* = 275 mV. The simulation results demonstrated strong agreement with the analytical model, as illustrated in Figure 10a. Minor deviations were observed at the triangle signal corners (at $90^{\circ }$, $180^{\circ }$, etc.) due to the model’s approximation at these points rather than the precise shape of the triangular waveform. The experimental results also aligned closely with the analytical model, as shown in Figure 11a, further validating the proposed approach.

#### 5.2.2. Triangular Amplitude Precision

The amplitude precision of the triangular signals was approximated from the analytical model developed in [22], with the modification factor shown in Equations (A3) and (A4) in Appendix A. The amplitude precision was experimentally measured as presented in Figure 11b, with an overall amplitude precision shift of 3 mV. The overall amplitude precision shift was likely associated with the electronic noise floor level of the system. However, further investigation is needed to confirm this. A maximum deviation of 0.5 mV in amplitude precision was observed, which was also the used ADC resolution limit.

#### 5.2.3. Triangular Phase

The triangular phase and phase precision can be approximated similarly to the derivation in [22] after segmenting the detection range into four sections. The triangular phase follows a non-linear relationship, as presented in Equation (A5) in Appendix A. Using the same approach, the phase precision follows Equation (A6) in Appendix A.

In the simulation results presented in Figure 10c, the phase error behaved consistently compared to the analytical model. The phase precision presented in Figure 10d also matches well with the analytical model, besides the peaks located at the triangle corners around $90^{\circ }$, $180^{\circ }$, and $270^{\circ }$. Such behavior could be suppressed using advanced phase detection algorithms.

The experimental results in Figure 11c show phase error oscillations due to the high ambient light compared to the limited integration length used. The maximum phase error detected peaks were +5.$38^{\circ }$ and −4.$79^{\circ }$, making the peak-to-peak phase error equal to 2.83% of the detection range. The phase precision presented in Figure 11d approximately matches the analytical model, besides some deviations due to the analytical model approximation. The phase precision could be reduced by utilizing multiple averaging stages or by increasing the integration length. The depth resolution was limited by the ADC used, as discussed in the discussion section in [21].

#### 5.2.4. Triangular and Sinusoidal Signal Comparison

**Figure 12:**
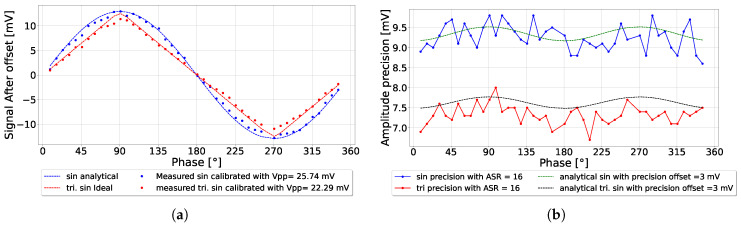

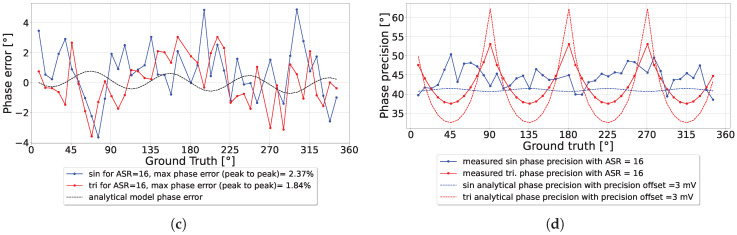
(**a**) Comparison between experimental measurements of triangular and sinusoidal amplitude with ASR = 16; (**b**) the amplitude precision; (**c**) the phase error; (**d**) the phase precision.

When experimentally applying an ASR of 16, the measured triangular and sinusoidal amplitudes were equally suppressed due to ambient rejection, as presented in Figure 12a. However, the triangular amplitude precision was lower than the sinusoidal amplitude precision by a factor of $\sqrt{2/3}$, as presented in Figure 12b. This behavior was attributed to the fact that the triangular input signals had a lower root mean squared (RMS) value compared to the sinusoidal signals. Therefore, the amplitude precision was seemingly lower in general compared to the sinusoidal precision. The phase error of both systems showed oscillation due to the high ambient light applied. When using triangular signals, the phase precision was worse at the corner points located at $90^{\circ }$, $180^{\circ }$, and $270^{\circ }$. Due to the lower amplitude detected compared to the sinusoidal signal, there was a slight influence on the phase precision.

## 6. Thoughts on Improvements in the Pixel Design

The multi-stage architecture proposed in Section 2 is promising, although many possible improvements could be thought of after the extensive analyses performed throughout this work. Some ideas will be proposed in this section, without providing the details. While doing so, one must keep in mind that there are always trade-offs to be made between simplicity, area, performance, and cost. As a reminder, the simplified schematic is shown in Figure 1a.

Beginning chronologically, the effects of timing jitter discussed in Section 3.1 were not directly observed in the measurements, but were surely present. There is the jitter due to the location of the electron–hole generation in the SPAD, and the additional jitter introduced due to the sensing circuitry. The technology used in the current pixel design implements the transistors in low-voltage NWELLs and PWELLs on a P-substrate, in which the SPAD anode is also directly implemented. In other words, there are no deep isolation wells, forcing the SPAD to be passively quenched and armed using an isolated high-voltage resistor and high-voltage AC coupling-capacitors for the sensing circuitry. Firstly, this increases the capacitance at the cathode and thus increases the jitter, while also increasing the afterpulsing probability. Secondly, as discussed in Section 3.2, this deteriorates the performance of the sensing circuit, especially at high intensity light levels, due to an increased probability of triggering at lower excess bias voltages during the arming of the SPAD.

A possible solution would be to use a more advanced technology with deep high-voltage isolation NWELLs, enabling the implementation of active quenching and arming transistors connected directly to the SPAD cathode, fixing both problems at once. However, this typically comes at the cost of large spacing constraints to ensure isolation, and thus at the cost of area and pixel density. Of course, this could be circumvented in a stacked technology, at the cost of more expensive technologies. To summarize, the sensing circuitry should be improved such that it still rejects afterpulsing and remains sensitive to any triggers past a certain threshold, without too much jitter and without variation in the output transient.

Concerning the deadtime shadowing discussed in Section 3.3, active arming and quenching once again provides a solution. In that case, you have the options to minimize the deadtime, or to intentionally delay the arming of the SPAD by one period. The first option is relatively simple to implement but does not fully mitigate the shadowing effect, thus influencing the confidence and phase of the measurement in somewhat unpredictable ways, depending on the scene and illumination, as discussed. The second option does not suffer from these problems, but comes at the cost of an increased settling time. An altogether different approach is to implement macro-pixels, each pixel with their own sampling circuit, but accumulating into a shared second stage. This solution would surely significantly improve the settling time, and likely significantly reduce the shadowing at high light intensities. Further investigation is required in this regard.

This brings us to Section 4.1 and Section 4.2, in which kTC noise and switching noise were discussed. To reduce such noise source, a possible solution would be to increase the capacitor size, at the expense of increasing the pixel size. This solution implies the switches need more time to fully transfer the charges, at least if their conductance remains unchanged. On the other hand, one trivial method for greatly diminishing the switching noise would be using transmission gates instead of only NMOS devices as switches. This would nevertheless complicate and enlarge the design significantly: not only the non-overlapping clocks for the sampling stage would now need to be generated in-pixel, but also their inverses, preferably with perfect alignment between the edges.

Regarding clock routing for multiple stages, the additional inverted clocks would need to be thoughtfully distributed across the array. This is a significant trade-off, but it resolves the charge injection and clock feedthrough, and guarantees good conductance for any input voltage. Alternatively, one could choose to implement dummy NMOS switches besides the NMOS switches used in the current implementation. This solution would, of course, not resolve the conductance issue, but it would mitigate the charge injection and clock feedthrough, while requiring less area compared to transmission gates. All this being said, the implementation of multiple stages remains essential, as demonstrated in Section 4.3.

The differential method proposed in Section 4.4 could possibly be further improved after a significant redesign. Currently, the in-phase measurement and the anti-phase measurement have to be performed sequentially, such that the low-frequency source follower errors are mostly compensated. One could adapt the circuit such that the two channels can be read out with one shared source follower alternating between them, enabling parallelization of these measurements. Special care should be taken not to introduce a significant new noise source due to this switching. Note that there would then also be a strong correlation between the in-phase measurement and the anti-phase measurement due to the simultaneous sampling, which was not the case previously. However, this leaves no channel for the quadrature measurement, meaning it would now need to be measured sequentially, losing the correlation instead.

## 7. Conclusions

This paper examined the systematic errors affecting the operation of the CA-dToF pixel, highlighting its robustness against noise sources such as SPAD timing jitter, kTC noise, and switching noise. Operational challenges, including deadtime shadowing effects, inertia effects, and source follower gain mismatch and offset, were also addressed. To mitigate these challenges, several improvements were proposed, including a macro-pixel implementation, differential measurement, multi-stage architectures, single source follower, and triangular signal implementation. The advantages and limitations of each solution were analyzed, providing insights into optimizing pixel performance.

Neglecting the proposed solutions for the CA-dToF pixel, the primary challenge affecting the phase accuracy is the source follower gain mismatch under low ambient light conditions and short deadtimes. The phase error oscillation, as discussed in Section 5.1, significantly impacts the phase accuracy. However, under high ambient light conditions, the influence of source follower gain mismatch on the pixel performance diminishes, and the dominant limitation becomes the deadtime shadowing effect.

An analytical model for the CA-dToF pixel utilizing a triangular signal was developed and evaluated through simulation and measurement. The triangular signal demonstrated reliable performance, with a maximum accuracy error of 2.81% of the detection range at an applied ASR of 54.9. The analysis deliberately excluded the detection range in terms of time, as the primary objective was to evaluate the pixel performance independently of the detection range. The experimental validation conducted in this study employed a sinusoidal signal with a frequency of 25 MHz. These findings can contribute to enhancing the reliability and robustness of CA-dToF systems for ToF applications.

## Data Availability

Data are contained within the article.

## Appendix A. Triangular Signal Formulas

### Appendix A.1

The appendix is an optional section that can contain details and data supplemental to the main text—for example, explanations of experimental details that would disrupt the flow of the main text but nonetheless remain crucial to understanding and reproducing the research shown; figures of replicates for experiments of which representative data are shown in the main text can be added here if brief, or as Supplementary Data. Mathematical proofs of results not central to the paper can be added as an appendix.

The expected signal amplitudes $E\left[{\hat{I}}_{tri}\right]\left(l\right)$ and $E\left[{\hat{Q}}_{tri}\right]\left(l\right)$ the following Equations:

(A1) $$\begin{array}{cccccccc} E\left[{\hat{I}}_{tri}\right]\left(l\right) & =\frac{S\cdot C\cdot a}{S\cdot a+A\cdot T}\frac{4\cdot l}{T} & \;0< & l\leq \frac{T}{4}-\frac{a}{2} & \;\; & =\frac{S\cdot C\cdot a}{S\cdot a+A\cdot T}\left(1-\frac{a}{T}\right) & \;\frac{T}{4}-\frac{a}{2}< & l\leq \frac{T}{4}+\frac{a}{2}\\ \; & =\frac{S\cdot C\cdot a}{S\cdot a+A\cdot T}\left(2-\frac{4\cdot l}{T}\right) & \;\frac{T}{4}+\frac{a}{2}< & l\leq \frac{3\cdot T}{4}-\frac{a}{2} & \;\; & =\frac{S\cdot C\cdot a}{S\cdot a+A\cdot T}\left(\frac{a}{T}-1\right) & \;\frac{3\cdot T}{4}-\frac{a}{2}< & l\leq \frac{3\cdot T}{4}+\frac{a}{2}\\ \; & =\frac{S\cdot C\cdot a}{S\cdot a+A\cdot T}\left(\frac{4\cdot l}{T}-4\right) & \;\frac{3\cdot T}{4}+\frac{a}{2}< & l\leq T \end{array}$$

(A2) $$\begin{array}{cccccccc} E\left[{\hat{Q}}_{tri}\right]\left(l\right) & =\frac{S\cdot C\cdot a}{S\cdot a+A\cdot T}\left(1-\frac{a}{T}\right) & \;T-\frac{a}{2}< & l\leq \frac{a}{2} & \;\; & =\frac{S\cdot C\cdot a}{S\cdot a+A\cdot T}\left(1-\frac{4\cdot l}{T}\right) & \;\frac{a}{2}< & l\leq \frac{T}{2}-\frac{a}{2}\\ \; & =\frac{S\cdot C\cdot a}{S\cdot a+A\cdot T}\left(\frac{a}{T}-1\right) & \;\frac{T}{2}-\frac{a}{2}< & l\leq \frac{T}{2}+\frac{a}{2} & \;\; & =\frac{S\cdot C\cdot a}{S\cdot a+A\cdot T}\left(\frac{4\cdot l}{T}-3\right) & \;\frac{T}{2}+\frac{a}{2}< & l\leq T-\frac{a}{2} \end{array}$$

The associated amplitude precisions $\sigma_{\hat{I_{tri}}}\left(l\right)$ and $\sigma_{\hat{Q_{tri}}}\left(l\right)$ are

(A3) $$\begin{array}{ll} \sigma_{\hat{I_{tri}}}{\left(l\right)}^{2} & =\frac{2\cdot L^{2}}{3\cdot (2n_{av}-1)}[\frac{A\cdot T\cdot C^{2}}{2(S\cdot a+A\cdot T)}+\frac{S\cdot C^{2}}{S\cdot a+A\cdot T}\frac{T}{2\pi }\left(\frac{\pi a}{T}-\frac{1}{2}\left(\cos\left(\frac{4\pi }{T}l\right)\sin\left(\frac{2\pi }{T}a\right)\right)\right)\\ & -{\left(\frac{S\cdot C}{S\cdot a+A\cdot T}\frac{T}{\pi }\left(\sin\left(\frac{2\pi }{T}l\right)\sin\left(\frac{\pi }{T}a\right)\right)\right)}^{2}], \end{array}$$

(A4) $$\begin{array}{ll} \sigma_{\hat{Q_{tri}}}{\left(l\right)}^{2} & =\frac{2\cdot L^{2}}{3\cdot (2n_{av}-1)}[\frac{A\cdot T\cdot C^{2}}{2(S\cdot a+A\cdot T)}+\frac{S\cdot C^{2}}{S\cdot a+A\cdot T}\frac{T}{2\pi }\left(\frac{\pi a}{T}+\frac{1}{2}\left(\cos\left(\frac{4\pi }{T}l\right)\sin\left(\frac{2\pi }{T}a\right)\right)\right)\\ & -{\left(\frac{S\cdot C}{S\cdot a+A\cdot T}\frac{T}{\pi }\left(\cos\left(\frac{2\pi }{T}l\right)\sin\left(\frac{\pi }{T}a\right)\right)\right)}^{2}]. \end{array}$$

The triangular signal phase is calculated using the following equation:

(A5) $$\theta =\left\{ \begin{array}{ll} \frac{T}{8}\left(\frac{\left|E\left[\hat{I_{tri}}\right](l)\right|-\left|E\left[\hat{Q_{tri}}\right](l)\right|}{\left|E\left[\hat{I_{tri}}\right](l)\right|+\left|E\left[\hat{Q_{tri}}\right](l)\right|}+1\right), & \mathrm{if}E\left[\hat{I_{tri}}\right](l)\geq 0\;\mathrm{and}\;E\left[\hat{Q_{tri}}\right](l))\geq 0\\ \frac{T}{8}\left(\frac{-\left|E\left[\hat{I_{tri}}\right](l)\right|+\left|E\left[\hat{Q_{tri}}\right](l)\right|}{\left|E\left[\hat{I_{tri}}\right](l)\right|+\left|E\left[\hat{Q_{tri}}\right](l)\right|}+3\right), & \mathrm{if}E\left[\hat{I_{tri}}\right](l)\geq 0\;\mathrm{and}\;E\left[\hat{Q_{tri}}\right](l))\leq 0\\ \frac{T}{8}\left(\frac{\left|E\left[\hat{I_{tri}}\right](l)\right|-\left|E\left[\hat{Q_{tri}}\right](l)\right|}{\left|E\left[\hat{I_{tri}}\right](l)\right|+\left|E\left[\hat{Q_{tri}}\right](l)\right|}+5\right), & \mathrm{if}E\left[\hat{I_{tri}}\right](l)\leq 0\;\mathrm{and}\;E\left[\hat{Q_{tri}}\right](l))\leq 0\\ \frac{T}{8}\left(\frac{-\left|E\left[\hat{I_{tri}}\right](l)\right|+\left|E\left[\hat{Q_{tri}}\right](l)\right|}{\left|E\left[\hat{I_{tri}}\right](l)\right|+\left|E\left[\hat{Q_{tri}}\right](l)\right|}+7\right), & \mathrm{if}E\left[\hat{I_{tri}}\right](l)\leq 0\;\mathrm{and}\;E\left[\hat{Q_{tri}}\right](l))\geq 0 \end{array}\right.$$

The analytical model approximation for the phase precision of the triangular signal is the following equation:

(A6) $$\sigma_{\theta }=\left\{\begin{array}{l} \sqrt{\begin{array}{ll} {\left(\frac{T}{8}\right)}^{2} & [{\left(\frac{1}{E\left[{\hat{I}}_{\mathrm{tri}}\right](l)+E\left[{\hat{Q}}_{\mathrm{tri}}\right](l)}+\frac{E\left[{\hat{Q}}_{\mathrm{tri}}\right](l)-E\left[{\hat{I}}_{\mathrm{tri}}\right](l)}{{\left(E\left[{\hat{I}}_{\mathrm{tri}}\right](l)+E\left[{\hat{Q}}_{\mathrm{tri}}\right](l)\right)}^{2}}\right)}^{2}\sigma_{\hat{I_{tri}}}(l{)}^{2}\\ & +{\left(-\frac{1}{E\left[{\hat{I}}_{\mathrm{tri}}\right](l)+E\left[{\hat{Q}}_{\mathrm{tri}}\right](l)}+\frac{E\left[{\hat{Q}}_{\mathrm{tri}}\right](l)-E\left[{\hat{I}}_{\mathrm{tri}}\right](l)}{{\left(E\left[{\hat{I}}_{\mathrm{tri}}\right](l)+E\left[{\hat{Q}}_{\mathrm{tri}}\right](l)\right)}^{2}}\right)}^{2}\sigma_{\hat{Q_{tri}}}(l{)}^{2}] \end{array}},\;\mathrm{if}\;E\left[{\hat{I}}_{\mathrm{tri}}\right]\left(l\right)\geq 0\;\;\mathrm{and}\;\;E\left[{\hat{Q}}_{\mathrm{tri}}\right]\left(l\right)\geq 0\\ \sqrt{\begin{array}{ll} {\left(\frac{T}{8}\right)}^{2} & [{\left(-\frac{1}{E\left[{\hat{I}}_{\mathrm{tri}}\right](l)-E\left[{\hat{Q}}_{\mathrm{tri}}\right](l)}+\frac{E\left[{\hat{Q}}_{\mathrm{tri}}\right](l)+E\left[{\hat{I}}_{\mathrm{tri}}\right](l)}{{\left(E\left[{\hat{I}}_{\mathrm{tri}}\right](l)-E\left[{\hat{Q}}_{\mathrm{tri}}\right](l)\right)}^{2}}\right)}^{2}\sigma_{\hat{I_{tri}}}(l{)}^{2}\\ & +{\left(-\frac{1}{E\left[{\hat{I}}_{\mathrm{tri}}\right](l)-E\left[{\hat{Q}}_{\mathrm{tri}}\right](l)}-\frac{E\left[{\hat{Q}}_{\mathrm{tri}}(l)+E\left[{\hat{I}}_{\mathrm{tri}}\right](l\right)}{{\left(E\left[{\hat{I}}_{\mathrm{tri}}\right](l)-E\left[{\hat{Q}}_{\mathrm{tri}}\right](l)\right)}^{2}}\right)}^{2}\sigma_{\hat{Q_{tri}}}(l{)}^{2}] \end{array}},\;\mathrm{if}\;E\left[{\hat{I}}_{\mathrm{tri}}\right]\left(l\right)\geq 0\;\;\mathrm{and}\;\;E\left[{\hat{Q}}_{\mathrm{tri}}\right]\left(l\right)\leq 0\\ \sqrt{\begin{array}{ll} {\left(\frac{T}{8}\right)}^{2} & [{\left(\frac{1}{E\left[{\hat{I}}_{\mathrm{tri}}\right](l)+E\left[{\hat{Q}}_{\mathrm{tri}}\right](l)}+\frac{E\left[{\hat{Q}}_{\mathrm{tri}}\right](l)-E\left[{\hat{I}}_{\mathrm{tri}}\right](l)}{{\left(E\left[{\hat{I}}_{\mathrm{tri}}\right](l)+E\left[{\hat{Q}}_{\mathrm{tri}}\right](l)\right)}^{2}}\right)}^{2}\sigma_{\hat{I_{tri}}}(l{)}^{2}\\ & +{\left(-\frac{1}{E\left[{\hat{I}}_{\mathrm{tri}}\right](l)+E\left[{\hat{Q}}_{\mathrm{tri}}\right](l)}+\frac{E\left[{\hat{Q}}_{\mathrm{tri}}\right](l)-E\left[{\hat{I}}_{\mathrm{tri}}\right](l)}{{\left(E\left[{\hat{I}}_{\mathrm{tri}}\right](l)+E\left[{\hat{Q}}_{\mathrm{tri}}\right](l)\right)}^{2}}\right)}^{2}\sigma_{\hat{Q_{tri}}}(l{)}^{2}] \end{array}},\;\mathrm{if}\;E\left[{\hat{I}}_{\mathrm{tri}}\right]\left(l\right)\leq 0\;\;\mathrm{and}\;\;E\left[{\hat{Q}}_{\mathrm{tri}}\right]\left(l\right)\leq 0\\ \sqrt{\begin{array}{ll} {\left(\frac{T}{8}\right)}^{2} & [{\left(\frac{1}{-E\left[{\hat{I}}_{\mathrm{tri}}\right](l)+E\left[{\hat{Q}}_{\mathrm{tri}}\right](l)}+\frac{E\left[{\hat{Q}}_{\mathrm{tri}}\right](l)+E\left[{\hat{I}}_{\mathrm{tri}}\right](l)}{{\left(-E\left[{\hat{I}}_{\mathrm{tri}}\right](l)+E\left[{\hat{Q}}_{\mathrm{tri}}\right](l)\right)}^{2}}\right)}^{2}\sigma_{\hat{I_{tri}}}(l{)}^{2}\\ & +{\left(\frac{1}{-E\left[{\hat{I}}_{\mathrm{tri}}\right](l)+E\left[{\hat{Q}}_{\mathrm{tri}}\right](l)}-\frac{E\left[{\hat{Q}}_{\mathrm{tri}}\right](l)+E\left[{\hat{I}}_{\mathrm{tri}}\right](l)}{{\left(-E\left[{\hat{I}}_{\mathrm{tri}}\right](l)+E\left[{\hat{Q}}_{\mathrm{tri}}\right](l)\right)}^{2}}\right)}^{2}\sigma_{\hat{Q_{tri}}}(l{)}^{2}] \end{array}},\;\mathrm{if}\;E\left[{\hat{I}}_{\mathrm{tri}}\right]\left(l\right)\leq 0\;\;\mathrm{and}\;\;E\left[{\hat{Q}}_{\mathrm{tri}}\right]\left(l\right)\geq 0 \end{array}\right.$$

## Appendix B. Sinusoidal Signal Formulas

As mentioned in [22], the analytical model provides the overall performance of the system at equilibrium, excluding the effect of the SPAD deadtime. The measured voltages $\hat{I}$ and $\hat{Q}$ have expected voltages $E\left[\hat{I}\right]\left(l\right)$ and $E\left[\hat{Q}\right]\left(l\right)$ equal to

(A7) $$\begin{array}{c} E\left[\hat{I}\right]\left(l\right)=\frac{S\cdot C}{S\cdot a+A\cdot T}\frac{T}{\pi }\left(\sin\left(\frac{2\pi }{T}l\right)\sin\left(\frac{\pi }{T}a\right)\right),\\ E\left[\hat{Q}\right]\left(l\right)=\frac{S\cdot C}{S\cdot a+A\cdot T}\frac{T}{\pi }\left(\cos\left(\frac{2\pi }{T}l\right)\sin\left(\frac{\pi }{T}a\right)\right). \end{array}$$

The confidence of the system at equilibrium is defined as the norm of the $\hat{I}$ and $\hat{Q}$:

(A8) $$\begin{array}{c} {\hat{C}}_{analytical}=\sqrt{{\hat{I}}^{2}+{\hat{Q}}^{2}}=\frac{S\cdot C}{S\cdot a+A\cdot T}\frac{T}{\pi }\sin\left(\frac{\pi }{T}a\right), \end{array}$$

where it can be approximated to ${\hat{C}}_{analytical}\approx \frac{S\cdot a}{S\cdot a+A\cdot T}C=\frac{1}{1+ASR}C,$ where $ASR=\frac{A\cdot T}{S\cdot a}$.

The variance in the detected voltages $\hat{I}$ and $\hat{Q}$ at equilibrium follows this equation:

(A9) $$\begin{array}{ll} \sigma_{\hat{I}}{\left(l\right)}^{2} & =\frac{L^{2}}{\left(2n_{av}-1\right)}[\frac{A\cdot T\cdot C^{2}}{2(S\cdot a+A\cdot T)}+\frac{S\cdot C^{2}}{S\cdot a+A\cdot T}\frac{T}{2\pi }\left(\frac{\pi a}{T}-\frac{1}{2}\left(\cos\left(\frac{4\pi }{T}l\right)\sin\left(\frac{2\pi }{T}a\right)\right)\right)\\ & -{\left(\frac{S\cdot C}{S\cdot a+A\cdot T}\frac{T}{\pi }\left(\sin\left(\frac{2\pi }{T}l\right)\sin\left(\frac{\pi }{T}a\right)\right)\right)}^{2}], \end{array}$$

(A10) $$\begin{array}{ll} \sigma_{\hat{Q}}{\left(l\right)}^{2} & =\frac{L^{2}}{\left(2n_{av}-1\right)}[\frac{A\cdot T\cdot C^{2}}{2(S\cdot a+A\cdot T)}+\frac{S\cdot C^{2}}{S\cdot a+A\cdot T}\frac{T}{2\pi }\left(\frac{\pi a}{T}+\frac{1}{2}\left(\cos\left(\frac{4\pi }{T}l\right)\sin\left(\frac{2\pi }{T}a\right)\right)\right)\\ & -{\left(\frac{S\cdot C}{S\cdot a+A\cdot T}\frac{T}{\pi }\left(\cos\left(\frac{2\pi }{T}l\right)\sin\left(\frac{\pi }{T}a\right)\right)\right)}^{2}]. \end{array}$$

The variance in the detected voltages affects the detected phase accuracy $E{\left[\theta \right]}_{a}nalytical\left(l\right)$, and variance $\sigma_{\theta \_analytical}\left(l\right)$ follows equations:

(A11) $$\begin{array}{ccc} E{\left[\theta \right]}_{analytical}\left(l\right) & \approx & \arctan\left(\frac{E[\hat{I}]}{E[\hat{Q}]}\right)+\frac{E\left[\hat{I}\right]\cdot E\left[\hat{Q}\right]}{{\left(E{\left[\hat{I}\right]}^{2}+E{\left[\hat{Q}\right]}^{2}\right)}^{2}}\left(\sigma_{\hat{Q}}^{2}-\sigma_{\hat{I}}^{2}\right) \end{array}$$

(A12) $$\begin{array}{ccc} \sigma_{\theta \_analytical}\left(l\right) & \approx & \left(\frac{E{\left[\hat{Q}\right]}^{2}}{{\left(E{\left[\hat{I}\right]}^{2}+E{\left[\hat{Q}\right]}^{2}\right)}^{2}}\sigma_{\hat{I}}^{2}+\frac{E{\left[\hat{I}\right]}^{2}}{{\left(E{\left[\hat{I}\right]}^{2}+E{\left[\hat{Q}\right]}^{2}\right)}^{2}}\sigma_{\hat{Q}}^{2}\right). \end{array}$$
